# Evaluating the Test Characteristics of a Prototype for AI-Assisted Radiographic Detection

**DOI:** 10.3390/dj14020096

**Published:** 2026-02-09

**Authors:** Rohit Kunnath Menon

**Affiliations:** Clinical Sciences Department, College of Dentistry, Ajman 3415, United Arab Emirates; r.menon@ajman.ac.ae

**Keywords:** artificial intelligence, AI, artificial intelligence in dentistry, dental AI

## Abstract

**Background/Objectives:** It is essential to test the accuracy of artificial intelligence-assisted tools that detect dental pathologies from radiographs. This study aimed to evaluate the test characteristics of an artificial intelligence-assisted convolutional neural network-based prototype used for automated radiographic detection. **Methods:** A total of 300 panoramic and 100 intraoral periapical radiographs were collected between January 2020 and 2024 and then analyzed by two trained, independent specialist evaluators. The diagnostic consensus, “ground truth”, was labeled as follows: BL: bone loss; C: caries; F: filling; I: implants; IT: impacted teeth; P: prosthesis; PC: post-core; PR: periapical radiolucency; RF: root fillings; and RR: retained roots. The radiographs were uploaded to the prototype, and the results were compared. Sensitivity, specificity, positive predictive value, and negative predictive value were calculated using Stata version 15.0 (StataCorp). **Results:** Overall, most of the outcomes demonstrated sensitivity greater than 82%, with values ranging from 66.41% (65.47,67.36) for BL to 100% (100.00,100.00) for I. For all outcomes, specificity was greater than 93%, with values ranging from 93.61% (93.12,94.10) for BL to 100% for I. The overall values for all the test characteristics for the periapical radiographs were above 85%. The key errors identified in the qualitative analysis were errors in tooth identification, failure to detect recurrent caries under fillings and crowns, impacted canines, and inaccurate identification of extensive fillings as crowns. **Conclusions:** The prototype demonstrated high sensitivity and specificity in identifying dental pathologies. Accuracy in identifying bone loss, teeth that have migrated, including impacted canines, secondary caries, and differentiating extensive fillings from crowns requires further improvement.

## 1. Introduction

The interpretation of dental radiographs is a critical element of clinical assessment in dentistry; however, the accuracy of these interpretations can differ greatly among clinicians, especially between those with limited experience and seasoned practitioners. Research has shown that undergraduate dental students often face difficulties in consistently analyzing radiographic images, underscoring the need for structured and tailored training programs to build strong diagnostic competence [1]. Such variability reinforces the importance of implementing decision-support systems that can help standardize radiographic evaluations and minimize practitioner-dependent inconsistencies.

Artificial intelligence (AI)—the ability of computer systems to perform tasks that typically require human cognitive functions, such as recognizing patterns, interpreting visual information, and making classifications—has become increasingly influential in medical imaging. Advanced AI models, particularly those based on deep learning and convolutional neural networks (CNNs), are capable of processing extensive imaging datasets with notable speed and accuracy [2]. In dentistry, these technologies are being used to streamline diagnostic workflows, lower associated costs, and assist clinicians with the initial interpretation of radiographic images [3,4]. Compared with earlier rule-based approaches, modern deep learning techniques automatically extract meaningful features from raw data, enhancing the reliability of image interpretation and object detection.

Panoramic radiographs are among the most commonly employed imaging tools in everyday dental practice due to the fact that they offer wide anatomical coverage, relatively low radiation exposure, and economic advantages [5,6,7,8,9]. These radiographs provide a broad view of maxillofacial structures and aid with the identification of conditions such as dental caries, periodontal bone defects, impacted teeth, and other abnormalities. Improvements in digital imaging technology have further increased the suitability of panoramic images for automated computational analysis [10,11].

Initial efforts to automate tasks such as tooth detection and numbering utilized manual pixel-based segmentation or traditional machine learning techniques, including support vector machines, feedforward networks, and sequence alignment methods [12,13]. These approaches, however, lacked the sophistication needed to distinguish subtle radiographic differences. Advances in CNN architectures and the incorporation of more refined image augmentation methods have significantly enhanced the accuracy and adaptability of AI models in dental imaging [14], contributing to the growth of interest in applying AI to panoramic radiograph interpretation.

A recent overview of systematic reviews investigating the applications of artificial intelligence in the analysis of dental panoramic radiographs has identified a significant body of research [15] examining the use of AI for detecting and categorizing a variety of dental and maxillofacial findings, with encouraging results reported in areas such as caries detection, assessment of periodontal bone loss, age estimation, and the identification of mandibular lesions, dental implants, and other structural anomalies. Despite these advancements, most previous studies focused on only one type of condition or a limited number of radiographic features. At present, a comprehensive evaluation of an AI-based system capable of identifying a wide range of clinically meaningful dental pathologies from panoramic images, including bone loss, caries, restorations, implants, impacted teeth, prostheses, posts and cores, periapical radiolucency, root fillings, and retained roots has not yet been performed.

This gap is particularly important because clinicians typically interpret multiple concurrent findings rather than isolated abnormalities. An AI system capable of detecting numerous conditions simultaneously could help standardize the diagnostic process, reduce missed findings, and serve as a training tool for students and less experienced practitioners. Although the field is expanding rapidly, few investigations have provided such an all-encompassing analysis or assessed the diagnostic performance of AI across a wide spectrum of dental pathologies.

To address this need, the present study examines the diagnostic performance of a prototype AI-based tool designed to automatically detect a comprehensive range of dental findings on panoramic radiographs. This study helps to advance the existing literature by (1) analyzing a broader set of radiographic conditions than previously explored, (2) testing the system using real clinical data, and (3) offering insights that may support future improvements and the integration of AI-driven diagnostic technologies in dental practice.

## 2. Materials and Methods

### 2.1. Study Design

A retrospective cross-sectional study design was employed. Ethical approval for this study was obtained from the Research Ethics Committee, Ajman University (D-F-H-28-Aug, 26 August 2024).

### 2.2. Sample Selection

The sample size was determined by assuming an expected average prevalence (P) of 0.8 and a precision level (d) of 0.05, as previously described by Bonfanti-Gris. [16]. To account for the potential coexistence of the target pathologies in a single radiograph and to enhance the statistical power of this study, a total of 300 radiographs were analyzed. Eight hundred panoramic radiographs, taken using the same machine and with the same exposure parameters, were collected between January 2020 and January 2024 from the College of Dentistry at Ajman University. Panoramic radiographs were obtained using a digital panoramic unit (Carestream CS 8100SC 3D, Carestream Dental, [USA]), operating within 73 kVp and 10 mA, with an exposure cycle of 11 s. Automatic exposure control was used, and the parameters were adjusted according to patient size. Intraoral periapical radiographs were taken using an intraoral X-ray unit operating at 65 kVp and 10 mA with an exposure time ranging from 0.40 to 0.60 s for adult patients depending on the tooth region.

### 2.3. Study Protocol

**Figure 1:**
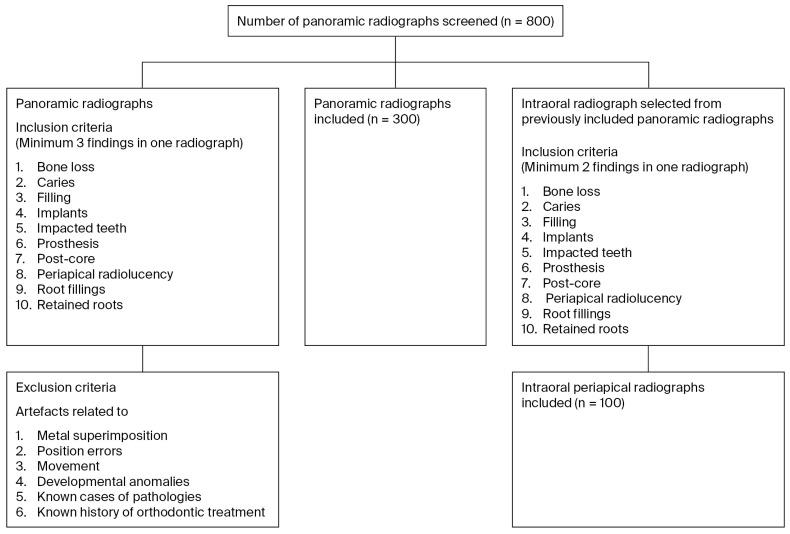
Flowchart for the selection of radiographs.

Initially, 800 panoramic radiographs were screened by a research assistant, and of these, 300 were included to ensure the presence of at least three identification parameters in one radiograph. The identification parameters are listed as follows: BL: bone loss; C: caries; F: filling; I: implants; IT: impacted teeth; P: prosthesis; PC: post-core; PR: periapical radiolucency; RF: root fillings; and RR: retained roots. Radiographs with artifacts related to metal superimposition, position errors, movement, developmental anomalies, known cases of pathologies, and known history of orthodontic treatment were excluded from this study. A corresponding intraoral periapical radiograph was selected from 100 randomly selected panoramic radiographs. It was ensured that each of the selected intraoral periapical radiographs had at least two of the included parameters. Two independent specialist evaluators with comparable clinical experience of more than 8 years (one specialist with postgraduate training and one with experience in the endodontic and pedodontic department) were trained in how to perform the radiographic identification by undergoing a calibration process. The flowchart for the selection process is depicted in Figure 1.

During the calibration process, twenty radiographs were carefully selected to ensure the presence of all the diagnostic criteria of interest. Each specialist entered the findings as per the coding described as follows: BL: bone loss; C: caries; F: filling; I: implants; IT: impacted teeth; P: prosthesis; PC: post-core; PR: periapical radiolucency; RF: root fillings; and RR: retained roots. After the entries were made in separate sheets, the research assistant screened them for any discrepancies. In the event of any discrepancy, a meeting was called for the specialist evaluators to discuss the disagreement and reach a diagnostic consensus. The diagnostic consensus was specified as the “ground truth”. If no agreement was reached between the two leading operators, a third reviewer evaluated the radiograph to achieve consensus among the evaluators. The radiograph was excluded if consensus could not be reached. Subsequently, using the methodology employed for the calibration process, a comprehensive analysis of all the selected radiographs was performed, as outlined previously. The existing dental structures were labeled with Federation Dentaire International (FDI) nomenclature according to the following categories: BL: bone loss; C: caries; F: filling; I: implants; IT: impacted teeth; P: prosthesis; PC: post-core; PR: periapical radiolucency; RF: root fillings; and RR: retained roots. To assess bone levels, each mesial and distal surface of a tooth was marked as having bone loss of less than 33% (bone less absent) and greater than 33% (bone loss present) via visual estimation. The selected periapical radiographs (100) were also assessed in a similar way. Subsequently, the research assistant uploaded the images to the prototype. The specialist evaluators were blinded to the AI output. No modifications were made to the quality of the radiograph to ensure consistency between the identification environment for the specialists and the prototype. The detection threshold of the program was adjusted to 0%, which corresponds to the percentage of trustworthiness with which the software indicates the presence of any dental structure or treatment. Thus, the prototype was able to identify the structures regardless of the reliability percentage obtained.

### 2.4. Statistical Analysis

The diagnostic sensitivity, specificity, positive predictive value (PPV), negative predictive value (NPV), receiver operating characteristic curve (ROC), and area under the ROC of the tested dataset were calculated using Stata version 15.0 (StataCorp) for statistical analysis [17]. Descriptive analysis was also performed. For the panoramic radiographs, sensitivity, specificity, PPV, and NPV results were generated for specific sections, including overall, maxillary anterior, maxillary posterior, mandibular anterior, and mandibular posterior regions, for all the following outcomes: BL, C, F, I, IT, P, PC, PR, RF, and RR. ChatGPT version 5.0 was used to generate the figure displaying the overall test characteristics for all outcomes. For the intraoral periapical radiographs, the overall sensitivity, specificity, PPV, and NPV for the identification of any outcome present were calculated. Outcome-specific analyses were not performed due to the insufficient number of cases for each individual outcome.

## 3. Results

**Figure 2:**
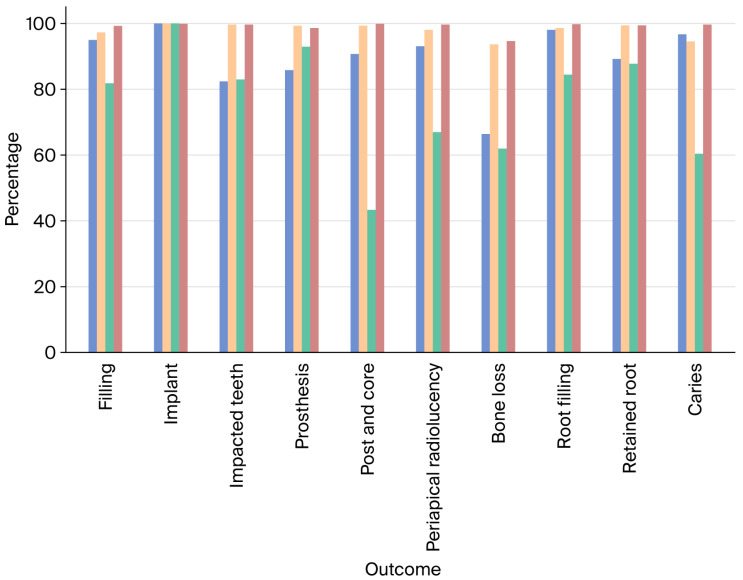
Test Characteristics (Overall) (In order: Sensitivity, Specificity, Positive Predictive Value, Negative Predictive Value). The confidence intervals for each value represented in the figure is provided in Table 1.

Overall, most of the outcomes demonstrated sensitivity greater than 82%, with values ranging from 66.41% for BL to 100% for I. For all outcomes, specificity was greater than 93%, with values ranging from 93.61% for BL to 100% for I. The overall values for all the test characteristics for the periapical radiographs were above 85%. The values for all the test characteristics across all the outcomes are provided in Figure 2.

For the maxillary anterior region, most of the outcomes demonstrated sensitivity greater than 90%, with values ranging from 66.67% for IT to 100% for I. For all outcomes, specificity was greater than 93%, with values ranging from 93.84% for C to 100% for I. For the maxillary posterior region, most of the outcomes demonstrated high sensitivity (>82%). The sensitivity values ranged from 68.65% for BL to 100% for I. For all outcomes, specificity was high (>95%). Furthermore, the specificity values ranged from 95.09% for BL to 100% for I. The values for the test characteristics across all the outcomes are provided in Table 1.

For the mandibular anterior region, most of the outcomes demonstrated high sensitivity (>83%). The sensitivity values ranged from 83.33% for PR to 100% for I. For all outcomes, specificity was high (>88%). Furthermore, the specificity values ranged from 88.26% for BL to 100% for I. For the mandibular posterior region, most of the outcomes demonstrated high sensitivity (>80%). The sensitivity values ranged from 53.16% for BL to 100% for I. For all outcomes, specificity was high (>93%). Moreover, the specificity values ranged from 93.45% for C to 100% for I.

The ROCs for all the outcomes across all the regions are provided in Appendix A.

The qualitative analysis of the radiographs revealed various challenges, as summarized in Table 2.

For the periapical radiographs, the prototype demonstrated the following values for the test characteristics [Sensitivity = 85.26% (81.44,88.56), Specificity = 98.79% (97.52,99.51), PPV = 98.02% (95.95,99.04), NPV = 90.51% (88.30,92.33).

## 4. Discussion

The prototype software demonstrated high sensitivity and specificity in detecting dental pathologies [18].

Previous studies have shown high sensitivity in detecting dental caries from radiographs, with reported sensitivity values ranging from 72.26% to 94.15% [19,20,21,22,23,24,25,26,27,28,29,30,31,32,33,34,35,36].

The identification of endodontic features, such as root canal fillings and post-cores, has demonstrated reasonable accuracy. However, as highlighted in recent research, evaluating the quality of root canal fillings still requires further training [37].

The prototype effectively identified the presence or absence of dental implants on radiographs. Nonetheless, additional training is needed to ensure that classification of implant types is accurate, success is predicted, and implant designs are optimized, consistent with the findings from prior studies that employed AI in implant dentistry [38,39,40,41,42,43,44].

Compared with previous investigations assessing periapical radiolucency, the prototype demonstrated similar sensitivity and improved specificity [45,46,47,48,49,50,51,52,53,54,55,56,57,58,59,60,61,62,63,64,65,66]. Notably, variations were observed among studies in terms of projection geometry, exposure parameters, film contrast, and film speed. Furthermore, differences in the radiographic data across studies must be acknowledged. These factors are crucial when interpreting pooled accuracy results from studies that use AI-based tools for detecting dental pathologies.

Sensitivity for detecting bone loss was generally lower than that for other outcomes. Prior deep learning-based studies have reported sensitivities ranging from 75% to 94%, with sample sizes varying from 100 to 2276 panoramic radiographs [66,67,68,69,70]. As per the revised periodontal disease classification, the criteria employed to determine radiographic bone loss are divided into three categories: <15%, 15–33%, and >33%. In the current study, the criteria were revised to include only two categories (<33% and >33%) to determine whether the prototype can dichotomously detect critical bone loss. Exact measurements were not made on the radiographs using digital measurement tools, as clinicians do not conventionally use accurate caliper-based measurements on each proximal root surface to determine the percentage of bone loss. Subjectivity may have also played a role when determining bone loss measurements. Additional refinement and training of the prototype are required to detect bone loss. However, radiographic bone loss on its own does not determine the clinical prognosis for the teeth, and so this must be supplemented with clinical measurements.

Sensitivity for identifying impacted teeth was lower compared with that for other outcomes. This may be attributed to the challenge of determining vertical impactions and also impactions with minimal mesial or distal tilt. However, a sensitivity greater than 80%, even in a diagnostically challenging clinical situation, may be considered acceptable. The identification of impacted teeth exclusively through radiographic evaluation may also be challenging for clinicians. Ideally, a combination of clinical and radiographic examinations must be performed [71]. Sensitivity for detecting impacted teeth was significantly lower in the maxillary anterior region, primarily due to the failure to detect impacted maxillary canines. The incidence of canine impactions is reported to be higher in the maxillary region than in the mandibular region, with a higher incidence reported in females [72]. The training of the prototype may have been primarily focused on detecting impacted teeth in the posterior region. Further training is required to include case types in the anterior region of the jaws, specifically canines and premolars.

The prototype found it challenging to differentiate extensive mesio-occluso-distal amalgam fillings from crowns. Extensive radio-opacity in the coronal region presents a challenge when it comes to distinguishing between the delineated margins of a crown and the less sharp margins of a filling. Further challenges in identifying cervical margins may be incorporated using cervical burnout. Training the prototype with additional images of an appropriate resolution may help to improve its accuracy.

For the periapical radiographs, an outcome-based evaluation was not performed owing to the limited sample size achieved for each outcome. The overall test characteristics were calculated based on the number of findings accurately identified when compared with the ground truth. The prototype was determined to have a high accuracy based on the test characteristics, with values above 85%. Periapical radiographs are more accurate for the definitive identification of caries, bone loss, and similar findings that require higher-resolution images [73]. Therefore, the prototype may help the clinician when cross-checking their findings and may even indicate conclusions that might be missed due to an intense working schedule.

For the current study, two specialists and a reviewer were employed to determine the diagnostic consensus, the “ground truth”. A similar approach has been used previously to establish the baseline truth in radiographic identification from dental radiographs [74,75]. Future validation of the prototype may involve incorporating additional reviewers for agreement, particularly in the domains of bone loss detection and tooth impaction. It is recommended that the reference standards for validating AI-assisted radiographic detection follow the established guidelines, and they can be approached in a stage-wise manner [76,77].

PPV and NPV are functions of sensitivity, specificity, and prevalence. In general, as disease prevalence increases, so does the PPV. The low prevalence of individual outcomes, compared with the total data points, which amount to more than 8000 individual teeth, may be the reason for the lower PPVs for some outcomes in the current study. In low-prevalence populations, the number of true positives will be small compared with the number of false positives, even if the test is highly accurate at distinguishing between those who do and do not have the disease. As a result, the PPV drops despite the high sensitivity and specificity.

It has been reported previously that AI tools show high diagnostic accuracy in identifying a variety of intraoral pathologies from two-dimensional radiographs [78]. Testing the diagnostic accuracy of each newly introduced tool is imperative to safeguard the enhancement of the efficiency of these tools as a support system for clinicians. Reductions in false positives and negatives pave the way for enhanced accuracy. It has been found that AI-assisted radiographic identification can improve the diagnostic accuracy of clinicians examining panoramic radiographs, resulting in a decrease in false positives [79]. Furthermore, AI tools are capable of improving the efficiency of the diagnostic process by increasing speed and highlighting potential findings [80].

The limitations of the tool used in this study may be attributed to selection bias with the radiographs used for the training of the model, which may limit its generalizability. However, this may only be substantiated by further testing in diverse clinical practices internationally. The prototype shows promise for applications in supplementing clinicians with limited experience and reducing errors. Furthermore, standardized use of the prototype across a practice with clinicians with varying levels of experience may result in taking a standardized approach to documentation, thus minimizing errors with medico-legal implications.

## 5. Conclusions

The dental prototype used in this study demonstrated high sensitivity and specificity in identifying dental pathologies. However, further improvements are required to enhance the accuracy of the tool when identifying teeth that have migrated, including impacted canines, secondary caries, and differentiating extensive fillings from crowns.

## Figures and Tables

**Table 1:** Test characteristics.

Outcome				
	Se (%)	Sp (%)	PPV (%)	NPV (%)
F (O)	95.05 (94.61, 95.48)	97.35 (97.03, 97.67)	81.82 (81.05, 82.59)	99.37 (99.21, 99.52)
F (MP)	94.51 (93.69, 95.32)	97.66 (97.12, 98.20)	87.32 (86.12, 88.51)	99.05 (98.70, 99.40)
F (MA)	92.00 (90.75, 93.25)	97.13 (96.35, 97.90)	80.00 (78.15, 81.85)	98.98 (98.52, 99.44)
F (MnP)	96.69 (96.05, 97.33)	96.36 (95.69, 97.03)	80.00 (78.57, 81.43)	99.49 (99.23, 99.74)
F (MnA)	100.0 (100.0, 100.0)	98.58 (98.03, 99.13)	61.54 (59.29, 63.79)	100.0 (100.0, 100.0)
I (O)	100.0 (100.0, 100.0)	100.0 (100.0, 100.0)	100.0 (100.0, 100.0)	100.0 (100.0, 100.0)
I (MP)	100.0 (100.0, 100.0)	100.0 (100.0, 100.0)	100.0 (100.0, 100.0)	100.0 (100.0, 100.0)
I (MA)	100.0 (100.0, 100.0)	100.0 (100.0, 100.0)	100.0 (100.0, 100.0)	100.0 (100.0, 100.0)
I (MnP)	100.0 (100.0, 100.0)	100.0 (100.0, 100.0)	100.0 (100.0, 100.0)	100.0 (100.0, 100.0)
I (MnA)	-	100.0 (100.0, 100.0)	-	-
IT (O)	82.43 (81.67, 83.19)	99.74 (99.63, 99.84)	82.99 (82.24, 83.74)	99.72 (99.62, 99.83)
IT (MP)	95.12 (94.35, 95.89)	99.70 (99.50, 99.89)	81.25 (79.85, 82.65)	99.93(99.84, 100.03)
IT (MA)	66.67 (64.49, 68.84)	99.33 (98.96, 99.71)	14.29 (12.67, 15.90)	99.94(99.83, 100.05)
IT (MnP)	77.88 (76.40, 79.37)	99.86 (99.73, 99.99)	95.29 (94.54, 96.05)	99.21 (98.89, 99.53)
IT (MnA)	-	100.0 (100.0, 100.0)	-	-
P (O)	85.85 (85.15, 86.55)	99.37 (99.21, 99.53)	93.03 (92.52, 93.54)	98.63 (98.39, 98.86)
P (MP)	84.23 (82.92, 85.53)	99.55 (99.31, 99.79)	95.70 (94.97, 96.42)	98.16 (97.68, 98.64)
P (MA)	94.79 (93.77, 95.82)	98.94 (98.47, 99.42)	91.46 (90.17, 92.75)	99.38 (99.01, 99.74)
P (MnP)	80.48 (79.06, 81.90)	99.56 (99.32, 99.79)	95.14 (94.37, 95.91)	97.93 (97.42, 98.44)
P (MnA)	92.59 (91.38, 93.80)	99.20 (98.79, 99.61)	78.13 (76.22, 80.03)	99.77 (99.55, 99.99)
PC (O)	90.74 (90.16, 91.32)	99.33 (99.17, 99.49)	43.36 (42.37, 44.35)	99.95 (99.90, 99.99)
PC (MP)	89.47 (88.38, 90.57)	99.36 (99.08, 99.65)	47.22 (45.44, 49.01)	99.93(99.84, 100.03)
PC (MA)	93.33 (92.18, 94.49)	98.82 (98.33, 99.32)	40.00 (37.74, 42.26)	99.94(99.83, 100.05)
PC (MnP)	90.00 (88.93, 91.07)	99.33 (99.04, 99.62)	47.37 (45.58, 49.16)	99.93(99.84, 100.03)
PC (MnA)	-	100.0 (100.0, 100.0)	-	-
PR (O)	93.06 (92.55, 93.57)	98.06 (97.78, 98.33)	66.91 (65.97, 67.85)	99.70 (99.59, 99.81)
PR (MP)	94.78 (93.99, 95.58)	97.12 (96.52, 97.72)	56.77 (55.00, 58.54)	99.79 (99.62, 99.95)
PR (MA)	100.0(100.0, 100.0)	98.37 (97.78, 98.95)	46.30 (43.99, 48.60)	100.00 (100.0, 100.0)
PR (MnP)	91.98 (91.01, 92.95)	97.90 (97.39, 98.41)	78.99 (77.53, 80.44)	99.30 (99.00, 99.60)
PR (MnA)	83.33 (81.61, 85.05)	99.50 (99.17, 99.82)	52.63 (50.32, 54.94)	99.89 (99.73, 100.04)
BL (O)	66.41 (65.47, 67.36)	93.61 (93.12, 94.10)	61.98 (61.01, 62.95)	94.67 (94.23, 95.12)
BL(MP)	68.65 (66.99, 70.31)	95.09 (94.32, 95.87)	76.05 (74.52, 77.58)	93.04 (92.13, 93.95)
BL (MA)	90.37 (89.01, 91.73)	94.05 (92.96, 95.15)	55.20 (52.91, 57.50)	99.18 (98.76, 99.59)
BL (MnP)	53.16 (51.38, 54.95)	95.51 (94.77, 96.25)	70.60 (68.97, 72.23)	90.95 (89.92, 91.98)
BL (MnA)	87.62 (86.10, 89.14)	88.26 (86.77, 89.75)	31.62 (29.47, 33.76)	99.14 (98.71, 99.57)
RF (O)	98.07 (97.80, 98.35)	98.63 (98.40, 98.87)	84.42 (83.69, 85.14)	99.85 (99.78, 99.93)
RF (MP)	97.25 (96.67, 97.84)	97.71 (97.18, 98.25)	82.03 (80.66, 83.40)	99.70 (99.50, 99.89)
RF (MA)	100.0 (100.0, 100.0)	98.64 (98.10, 99.17)	83.33 (81.61, 85.05)	100.0 (100.0, 100.0)
RF (MnP)	97.97 (97.46, 98.47)	98.98 (98.62, 99.34)	89.59 (88.50, 90.68)	99.82 (99.66, 99.97)
RF (MnA)	100.0 (100.0, 100.0)	99.49 (99.17, 99.82)	70.97 (68.87, 73.06)	100.0 (100.0, 100.0)
RR (O)	89.18 (88.55, 89.80)	99.42 (99.27, 99.57)	87.73 (87.08, 88.39)	99.50 (99.36, 99.64)
RR (MP)	92.17 (91.20, 93.13)	99.28 (98.98, 99.58)	90.91 (89.88, 91.94)	99.39 (99.11, 99.67)
RR (MA)	92.50 (91.28, 93.72)	99.60 (99.31, 99.89)	84.09 (82.40, 85.78)	99.83 (99.64, 100.0)
RR (MnP)	84.38 (83.08, 85.67)	99.15 (98.83, 99.48)	84.91 (83.62, 86.19)	99.12 (98.79, 99.45)
RR (MnA)	87.50 (85.97, 89.03)	99.89 (99.73, 100.04)	77.78 (75.86, 79.70)	99.94 (99.84, 100.05)
C (O)	96.72 (96.36, 97.08)	94.52 (94.07, 94.98)	60.36 (59.38, 61.34)	99.70 (99.59, 99.81)
C (MP)	96.64 (95.99, 97.28)	95.32 (94.57, 96.08)	71.66 (70.04, 73.27)	99.57 (99.34, 99.80)
C (MA)	97.50 (96.78, 98.22)	93.84 (92.73, 94.95)	42.39 (40.11, 44.67)	99.88 (99.71, 100.04)
C (MnP)	96.33 (95.66, 97.00)	93.45 (92.57, 94.34)	64.29 (62.57, 66.00)	99.52 (99.28, 99.77)
C (MnA)	100.0 (100.0, 100.0)	95.60 (94.65, 96.55)	26.42 (24.38, 28.45)	100.0 (100.0, 100.0)

BL: bone loss; C: caries; F: filling; I: implants; IT: impacted teeth; NPV: negative predictive value; P: prosthesis; PC: post-core; PR: periapical radiolucency; PPV: positive predictive value; RF: root fillings; RR: retained roots; Se: sensitivity; Sp: specificity.

**Table 2:** Qualitative assessment of challenges.

Domain	Finding
Cervical caries	Failure to identify caries in the proximal cervical region of a tooth.
Recurrent caries	Failure to identify recurrent caries underneath restorations and crowns.
Tooth identification	Failure to identify tooth that has migrated to the position of another tooth after extraction.
Tooth number identification	Failure to identify the correct tooth number when multiple retained roots are present.
Bone loss	Failure to accurately identify bone loss with crowding and the presence of multiple crowns and bridges.
Extensive restorations	Identification of extensive mesio-occlusal-distal fillings as crowns.
Prosthesis	Failure to identify veneers with diminished radio-opacity.
Large carious lesion	Identification of large carious lesions as retained roots.
Caries and bone loss	Identification of caries as bone loss when it is present at the cervical region.
Root caries	Failure to identify root caries.
Impacted teeth	Failure to detect impacted canines and premolars.
Retained roots	Failure to detect isolated retained roots on the surface.
Periapical radiolucency	Failure to detect mild periapical involvement.
Post-core	Failure to detect fiber posts.

**Figure A1:**
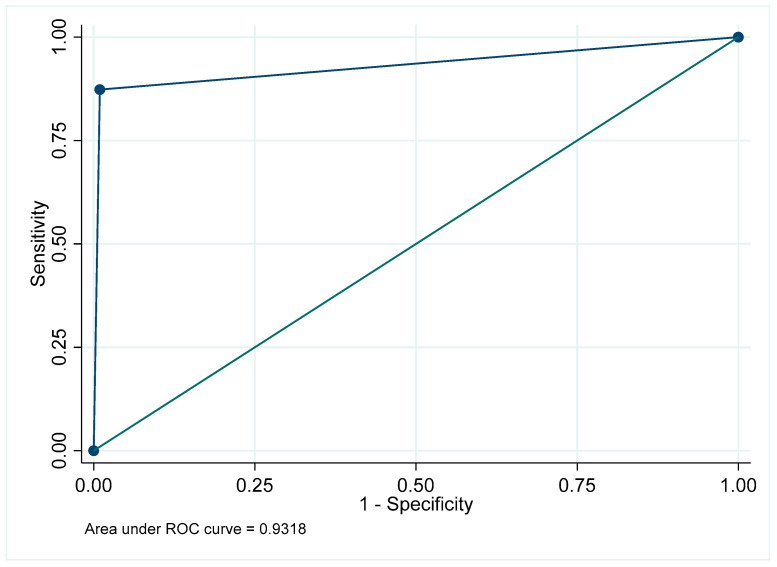
Receiver Operating Characteristic curve for filling in the maxillary posterior region.

**Figure A2:**
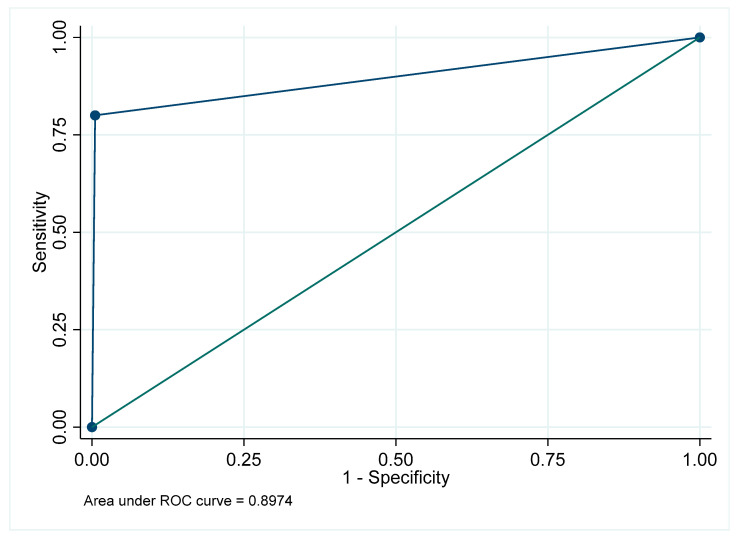
Receiver Operating Characteristic curve for filling in the mandibular posterior region.

**Figure A3:**
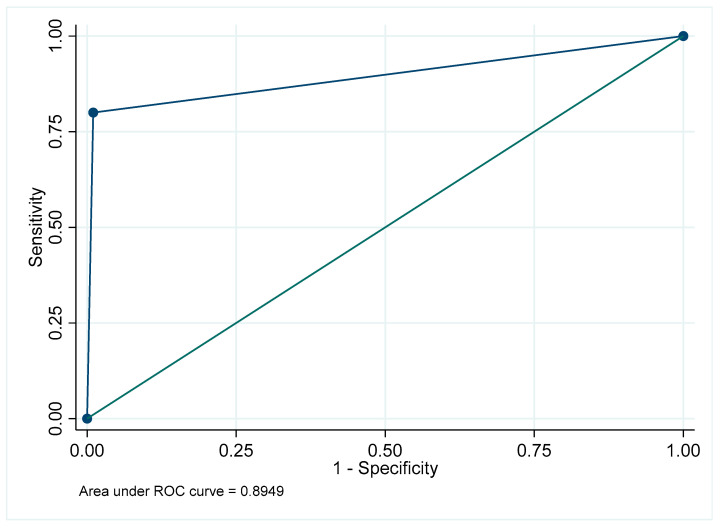
Receiver Operating Characteristic curve for filling in the maxillary anterior region.

**Figure A4:**
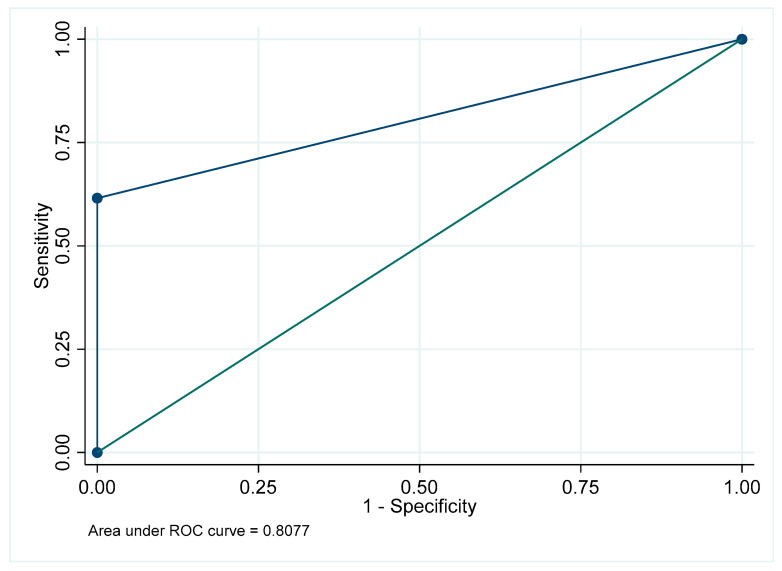
Receiver Operating Characteristic curve for filling in the mandibular anterior region.

**Figure A5:**
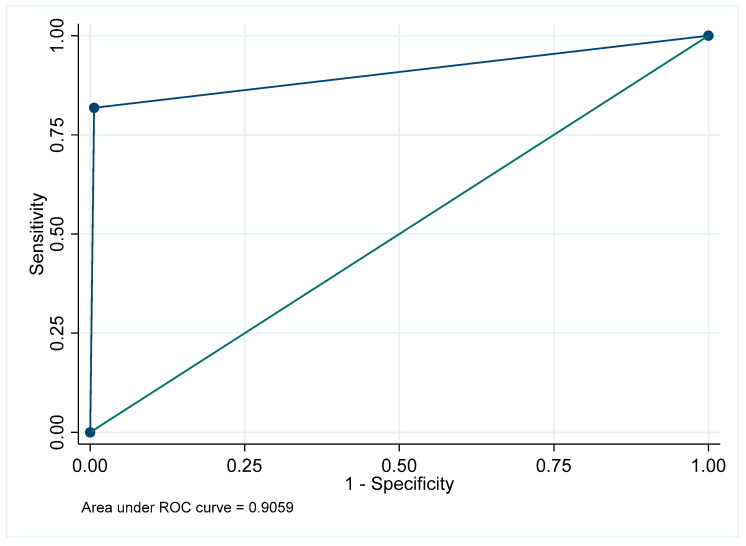
Receiver Operating Characteristic curve for filling: Overall.

**Figure A6:**
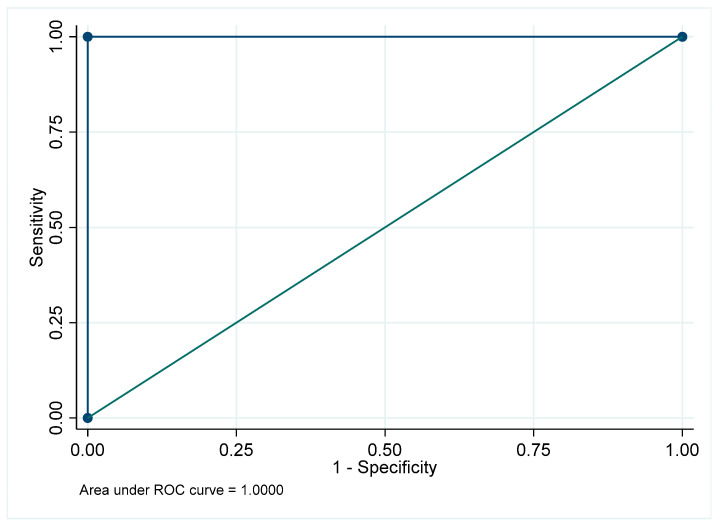
Receiver Operating Characteristic curve for implants in the maxillary posterior region.

**Figure A7:**
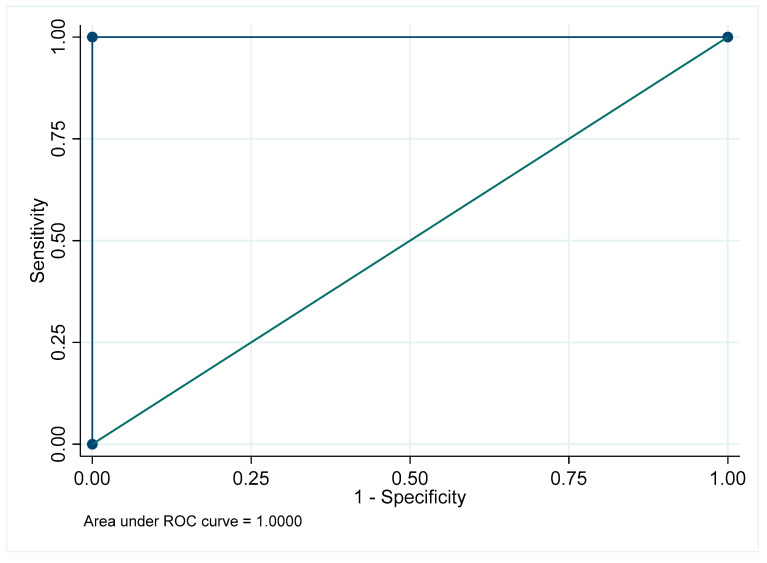
Receiver Operating Characteristic curve for implants in the mandibular posterior region.

**Figure A8:**
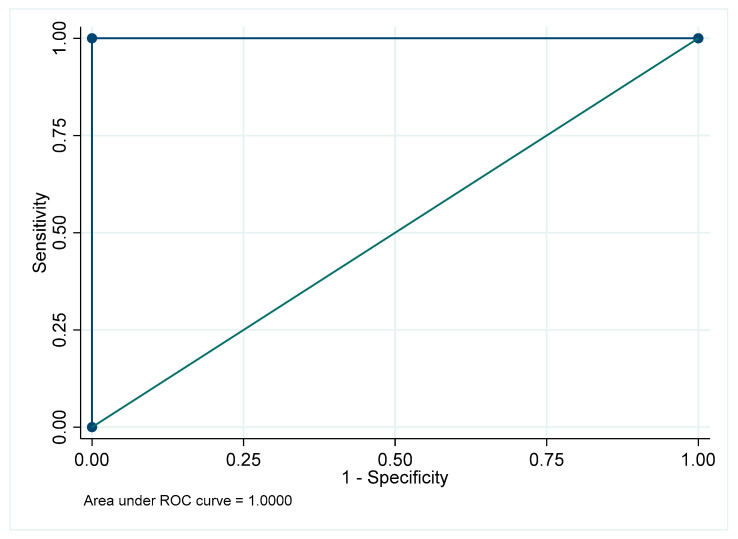
Receiver Operating Characteristic curve for implants in the maxillary anterior region.

**Figure A9:**
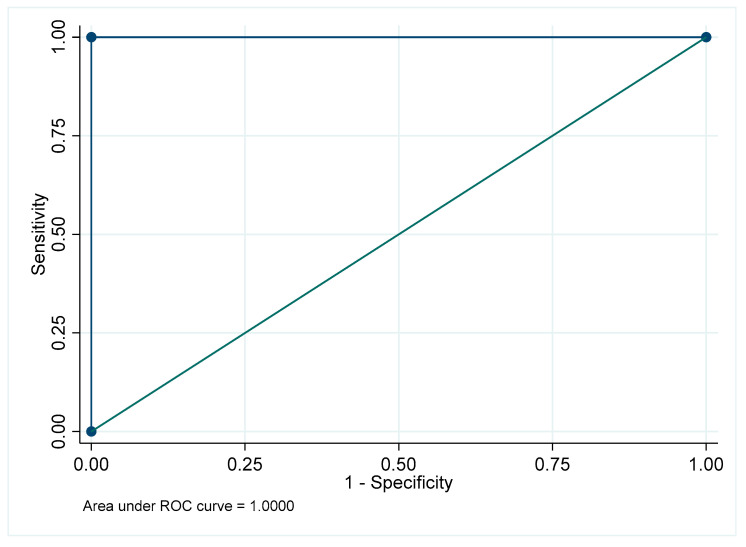
Receiver Operating Characteristic curve for implants: Overall.

**Figure A10:**
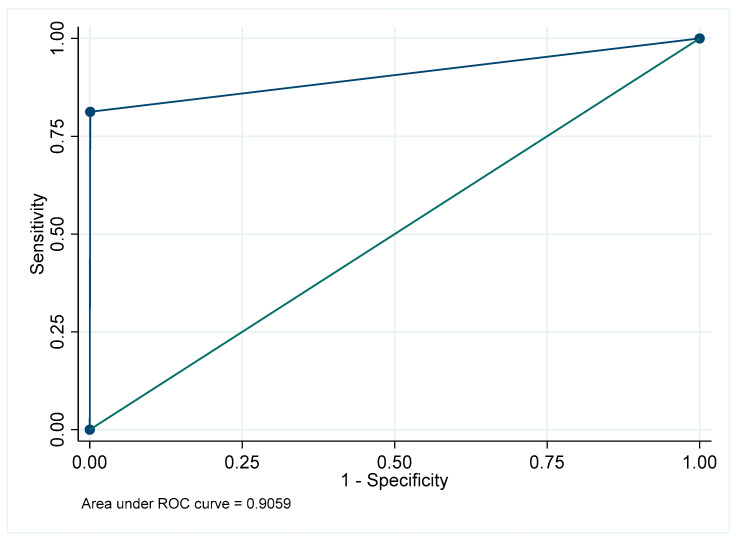
Receiver Operating Characteristic curve for impacted teeth in the maxillary posterior region.

**Figure A11:**
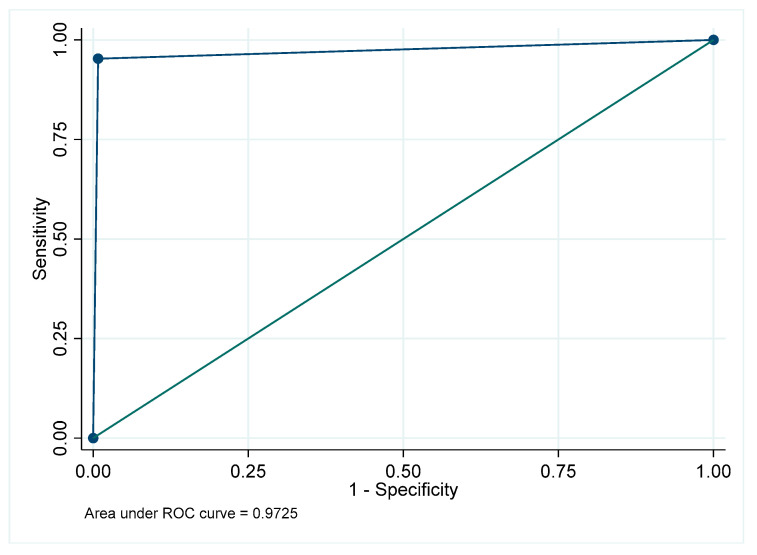
Receiver Operating Characteristic curve for impacted teeth in the mandibular posterior region.

**Figure A12:**
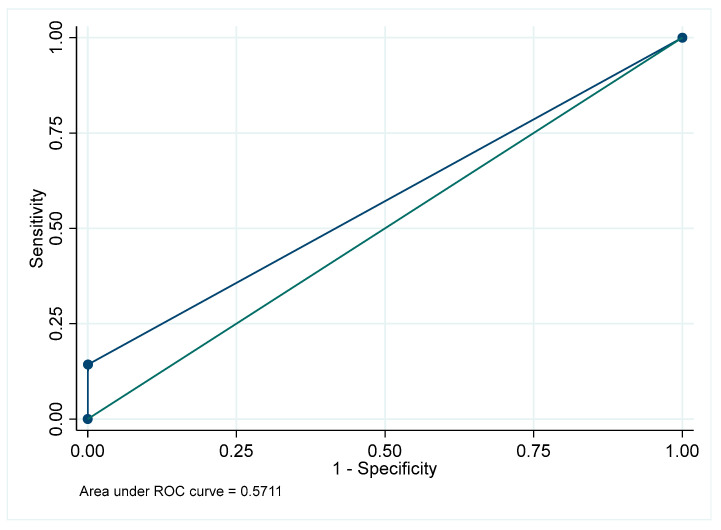
Receiver Operating Characteristic curve for impacted teeth in the maxillary anterior region.

**Figure A13:**
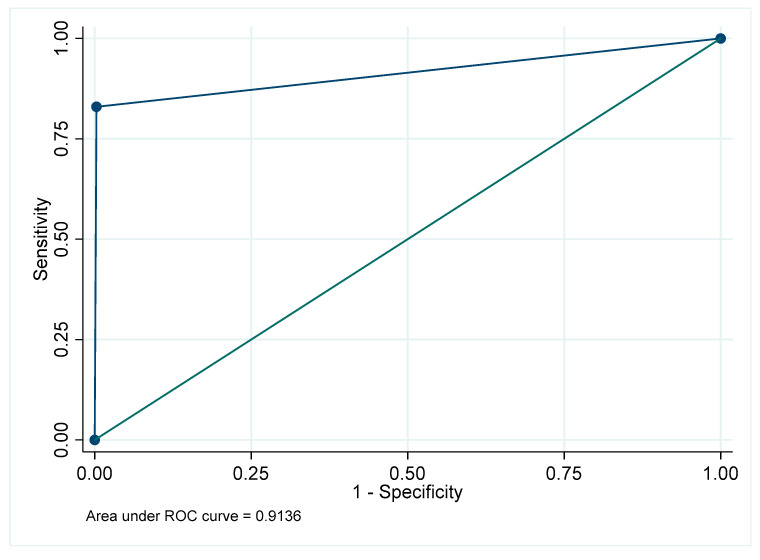
Receiver Operating Characteristic curve for impacted teeth: Overall.

**Figure A14:**
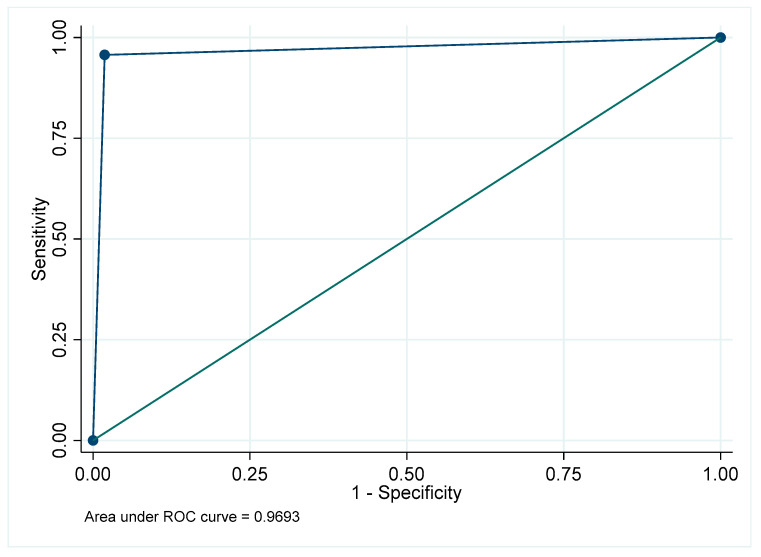
Receiver Operating Characteristic curve for prosthesis in the maxillary posterior region.

**Figure A15:**
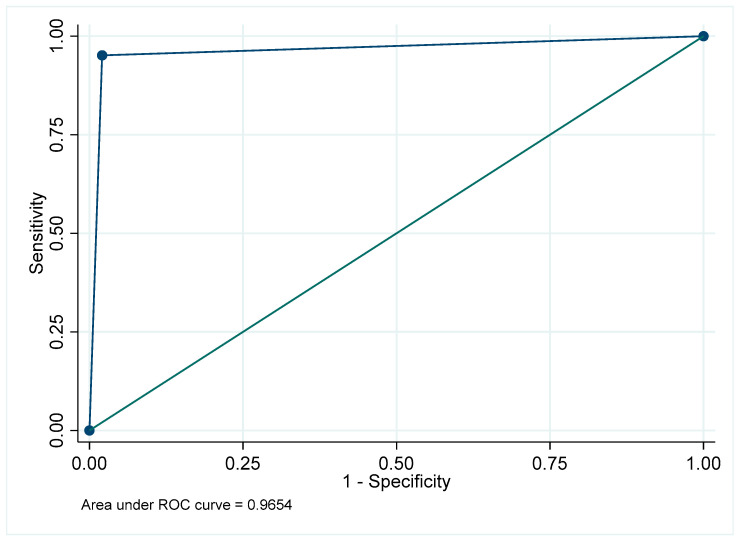
Receiver Operating Characteristic curve for prosthesis in the mandibular posterior region.

**Figure A16:**
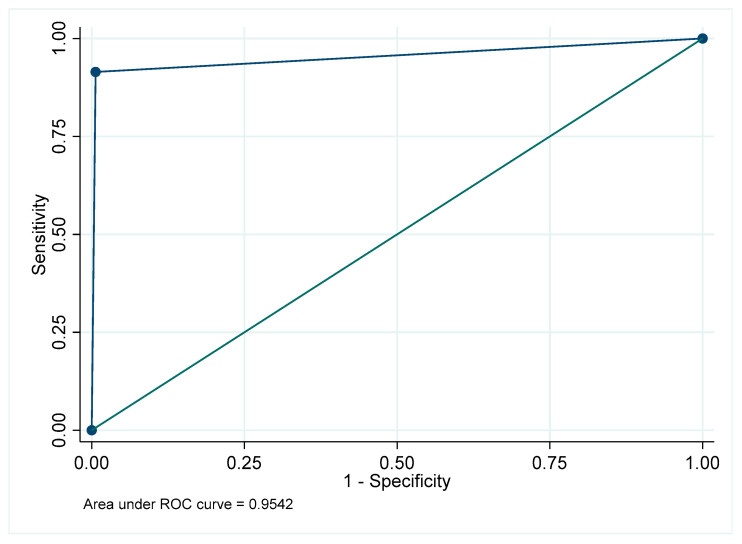
Receiver Operating Characteristic curve for prosthesis in the maxillary anterior region.

**Figure A17:**
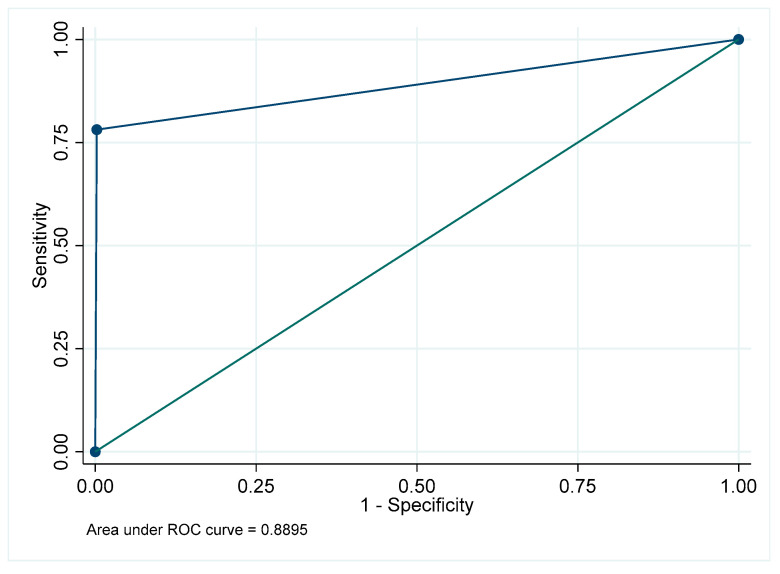
Receiver Operating Characteristic curve for prosthesis in the mandibular anterior region.

**Figure A18:**
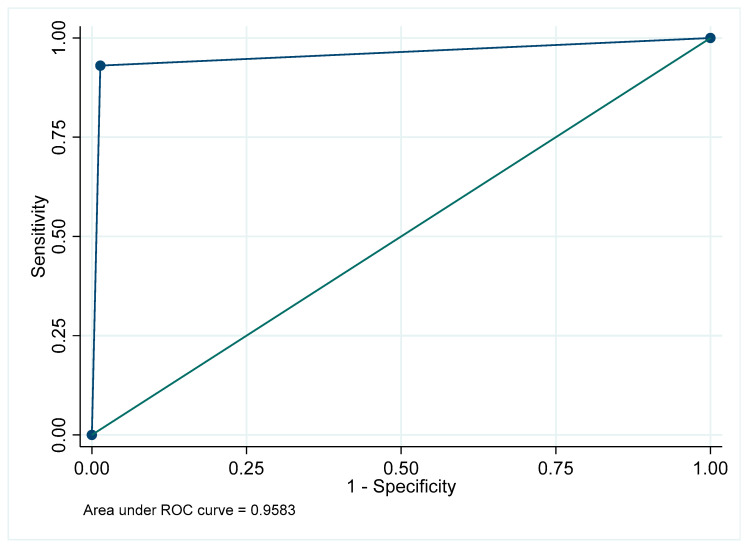
Receiver Operating Characteristic curve for prosthesis: Overall.

**Figure A19:**
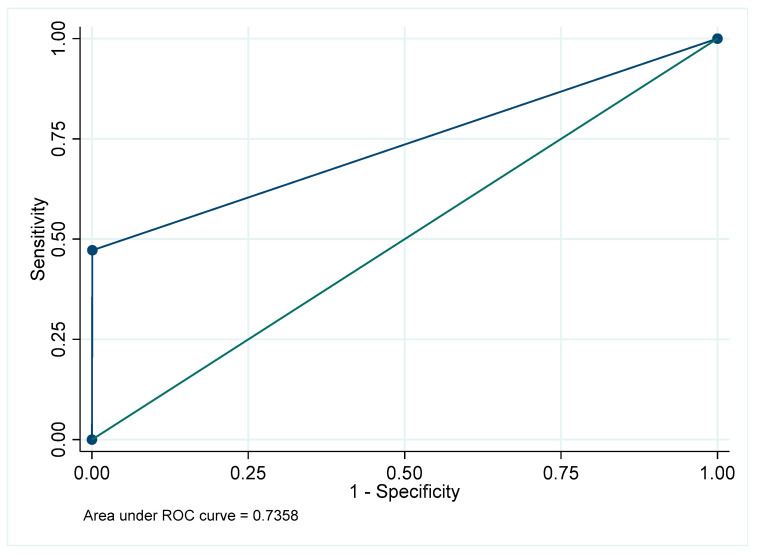
Receiver Operating Characteristic curve for post and core in the maxillary posterior region.

**Figure A20:**
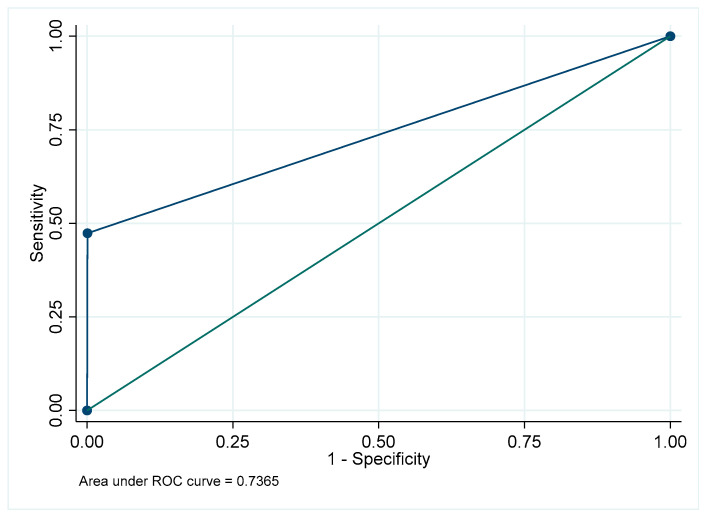
Receiver Operating Characteristic curve for post and core in the mandibular posterior region.

**Figure A21:**
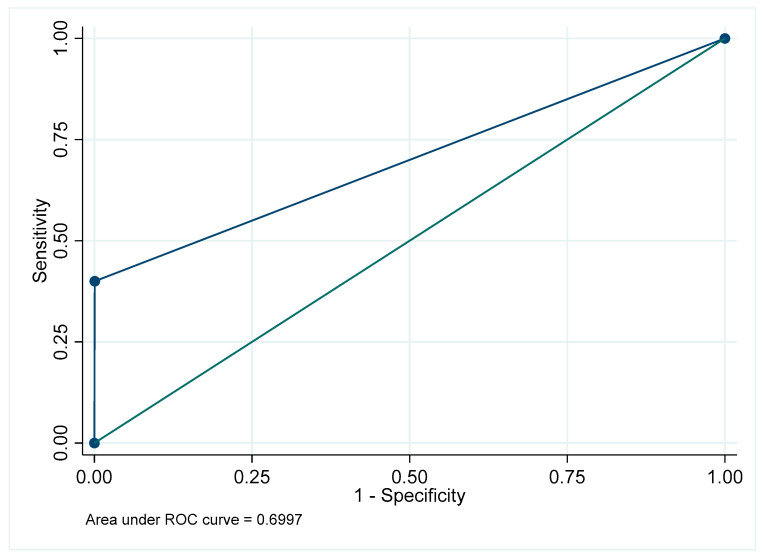
Receiver Operating Characteristic curve for post and core in the maxillary anterior region.

**Figure A22:**
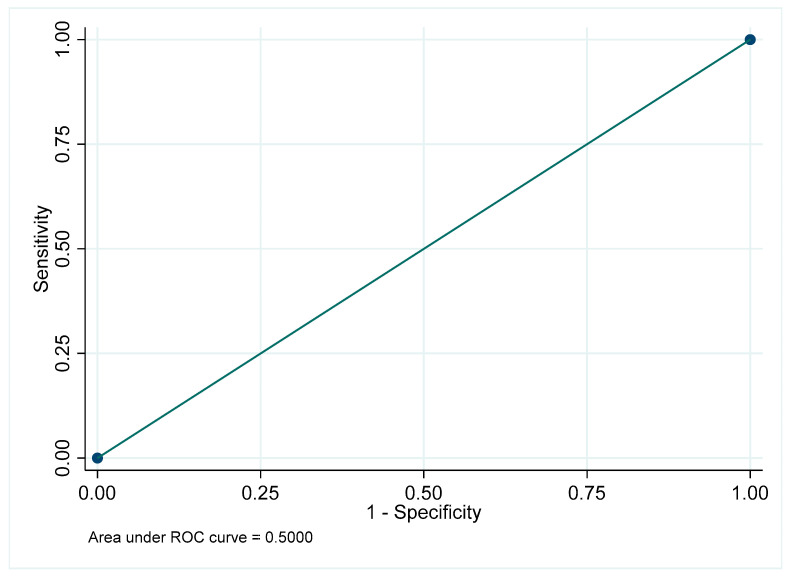
Receiver Operating Characteristic curve for post and core in the mandibular anterior region.

**Figure A23:**
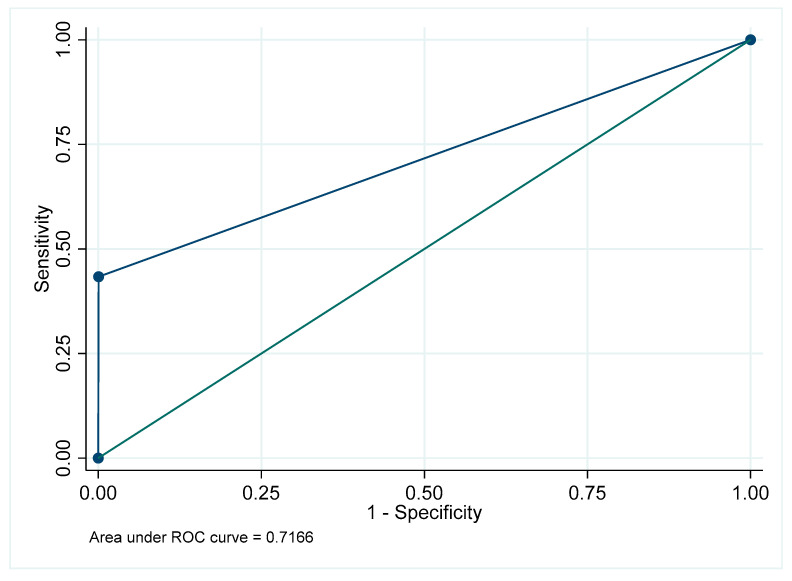
Receiver Operating Characteristic curve for post and core: Overall.

**Figure A24:**
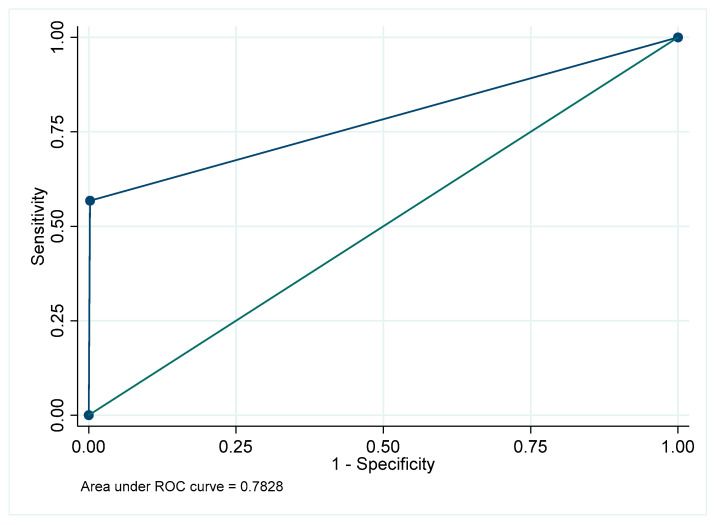
Receiver Operating Characteristic curve for periapical radiolucency in the maxillary posterior region.

**Figure A25:**
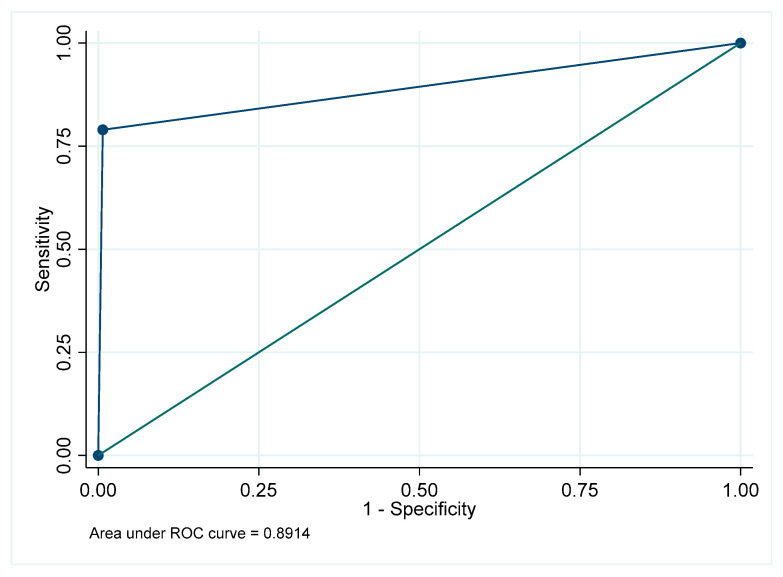
Receiver Operating Characteristic curve for periapical radiolucency in the mandibular posterior region.

**Figure A26:**
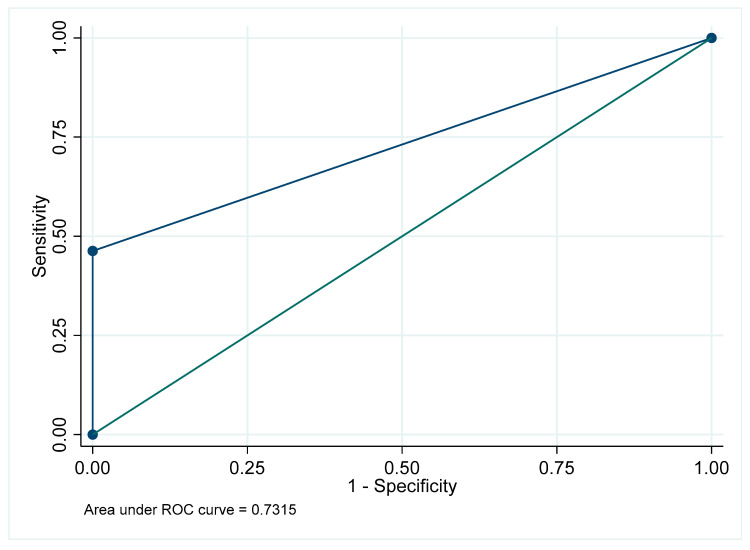
Receiver Operating Characteristic curve for periapical radiolucency in the maxillary anterior region.

**Figure A27:**
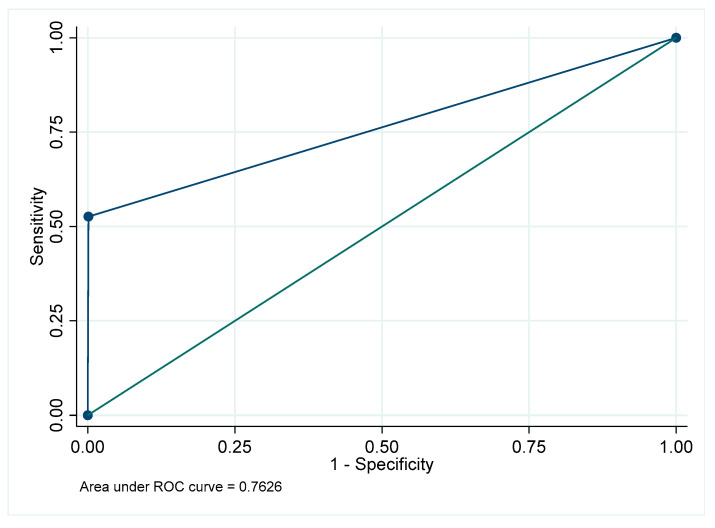
Receiver Operating Characteristic curve for periapical radiolucency in the mandibular anterior region.

**Figure A28:**
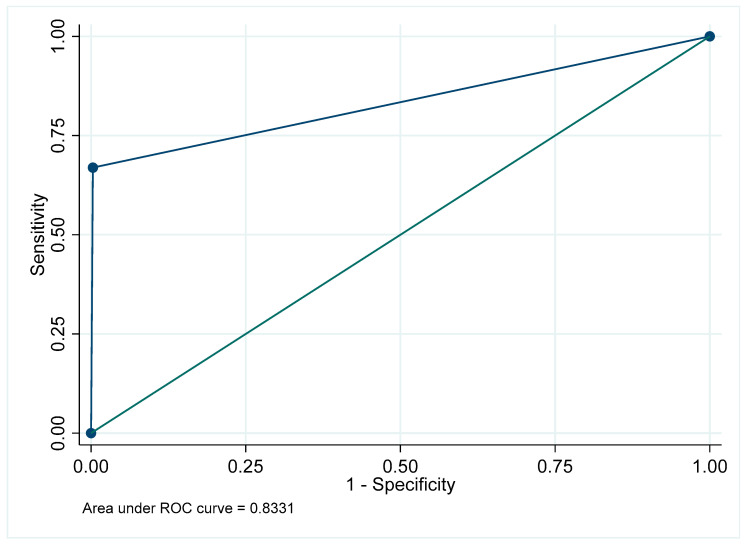
Receiver Operating Characteristic curve for periapical radiolucency: Overall.

**Figure A29:**
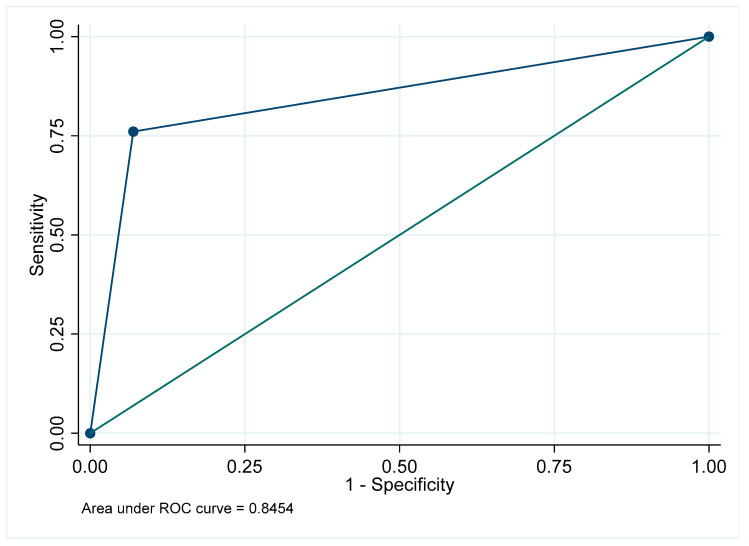
Receiver Operating Characteristic curve for bone loss in the maxillary posterior region.

**Figure A30:**
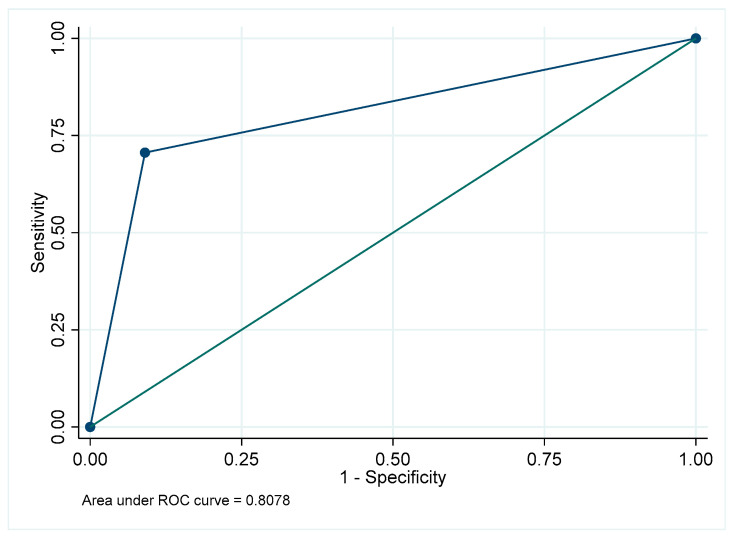
Receiver Operating Characteristic curve for bone loss in the mandibular posterior region.

**Figure A31:**
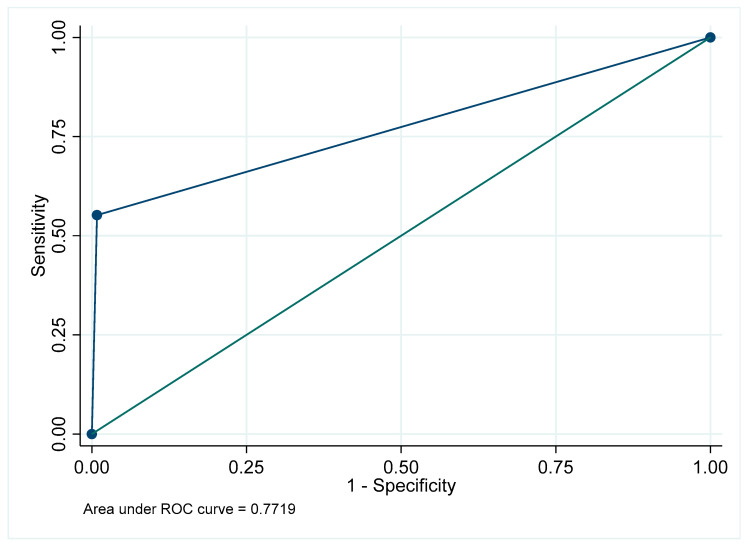
Receiver Operating Characteristic curve for bone loss in the maxillary anterior region.

**Figure A32:**
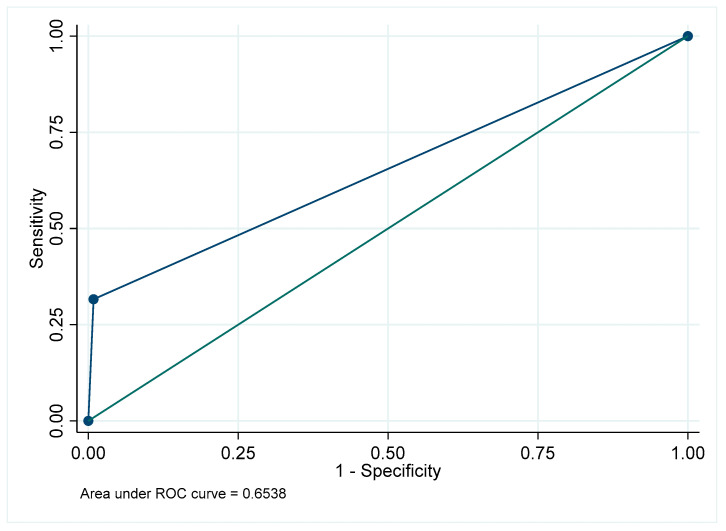
Receiver Operating Characteristic curve for bone loss in the mandibular anterior region.

**Figure A33:**
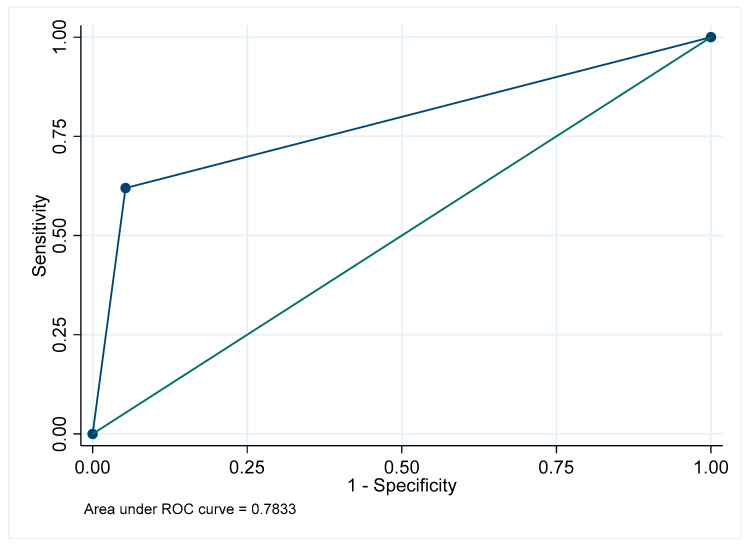
Receiver Operating Characteristic curve for bone loss: Overall.

**Figure A34:**
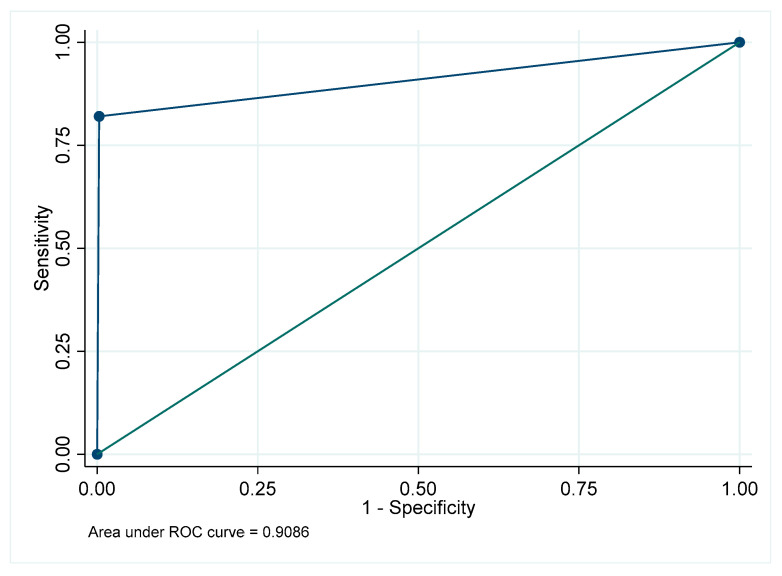
Receiver Operating Characteristic curve for root fillings in the maxillary posterior region.

**Figure A35:**
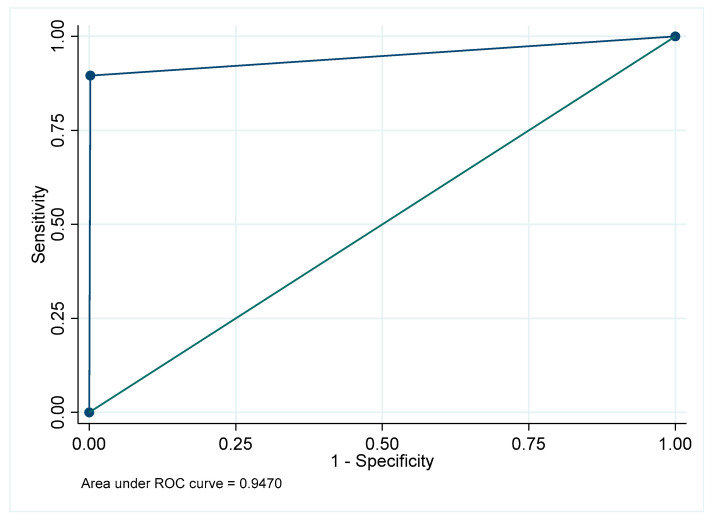
Receiver Operating Characteristic curve for root fillings in the mandibular posterior region.

**Figure A36:**
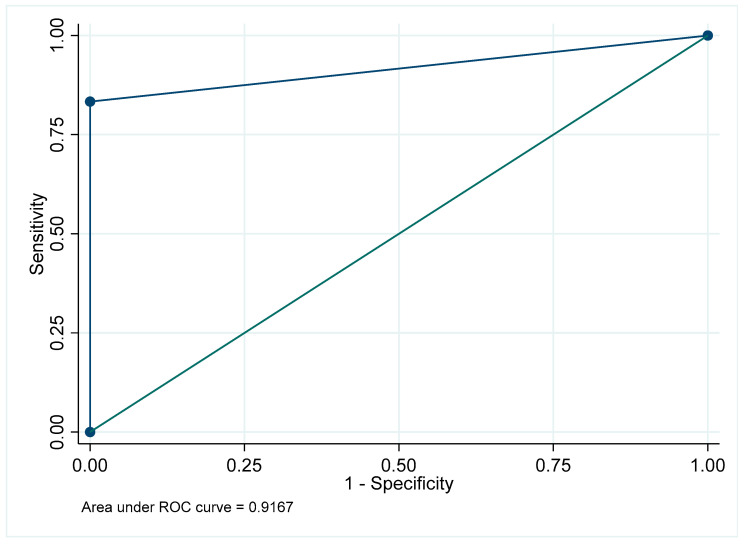
Receiver Operating Characteristic curve for root fillings in the maxillary anterior region.

**Figure A37:**
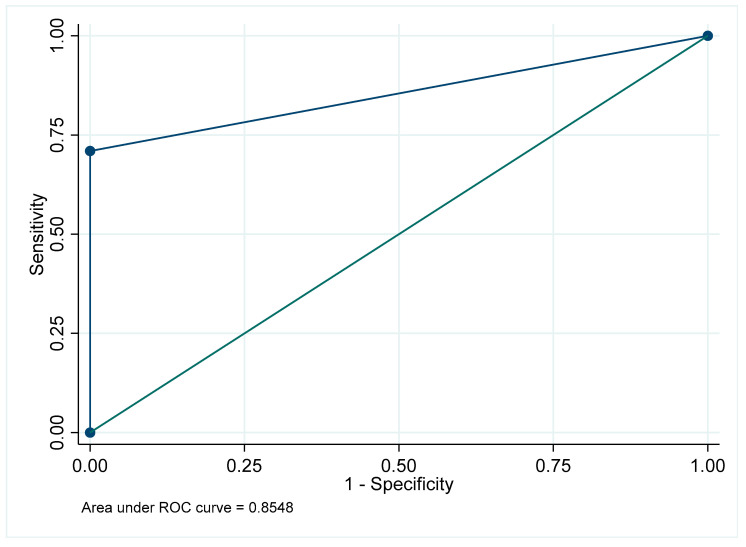
Receiver Operating Characteristic curve for root fillings in the mandibular anterior region.

**Figure A38:**
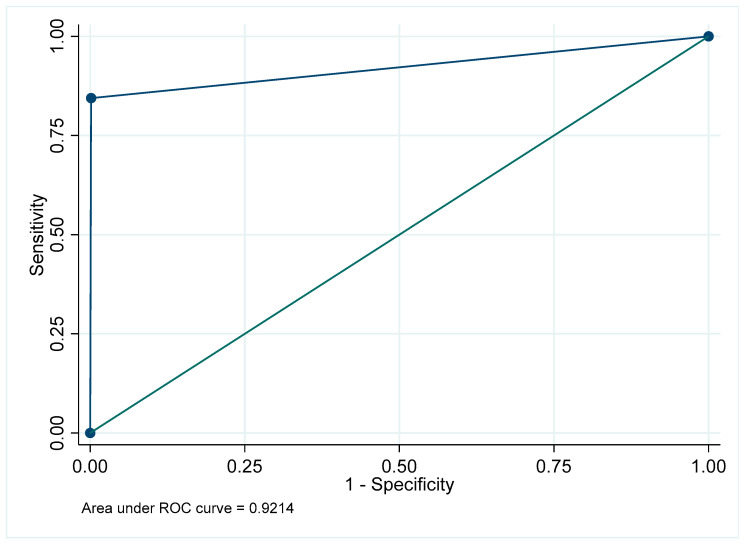
Receiver Operating Characteristic curve for root fillings: Overall.

**Figure A39:**
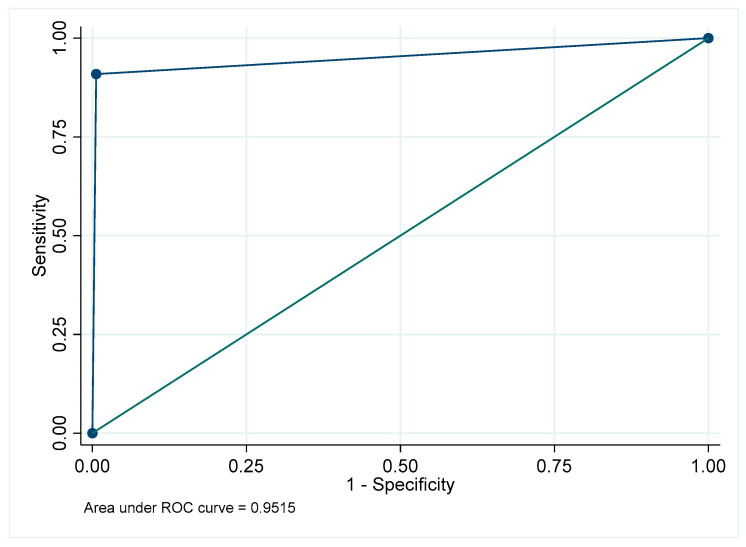
Receiver Operating Characteristic curve for retained root in the maxillary posterior region.

**Figure A40:**
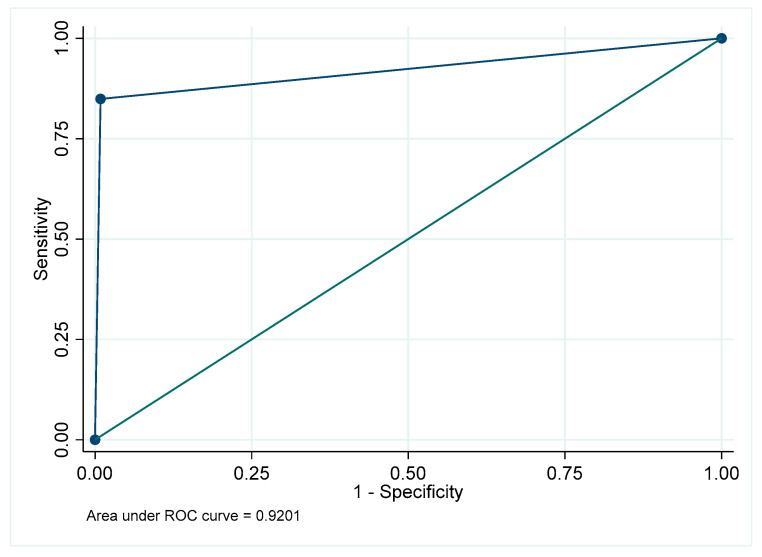
Receiver Operating Characteristic curve for retained root in the mandibular posterior region.

**Figure A41:**
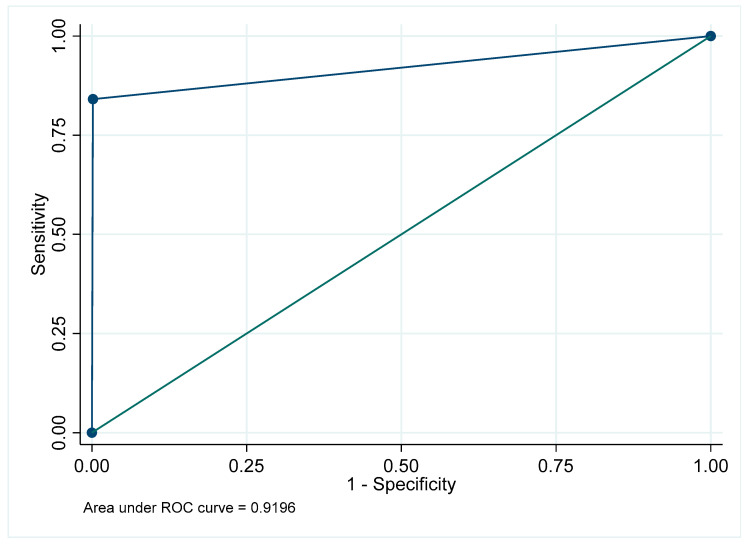
Receiver Operating Characteristic curve for retained root in the maxillary anterior region.

**Figure A42:**
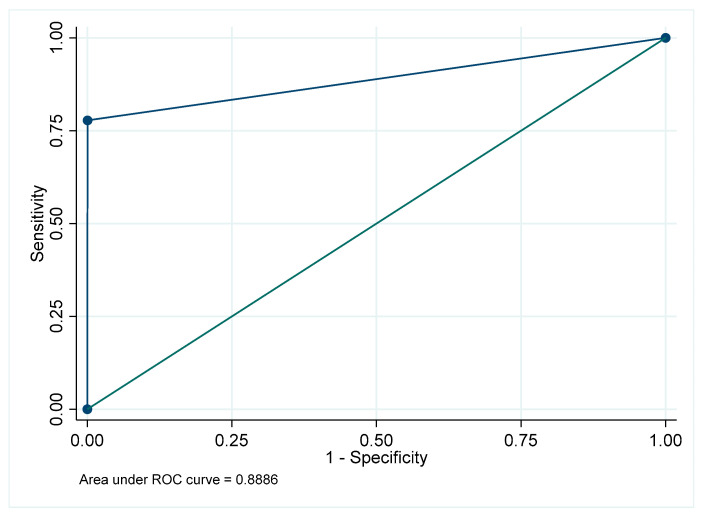
Receiver Operating Characteristic curve for retained root in the mandibular anterior region.

**Figure A43:**
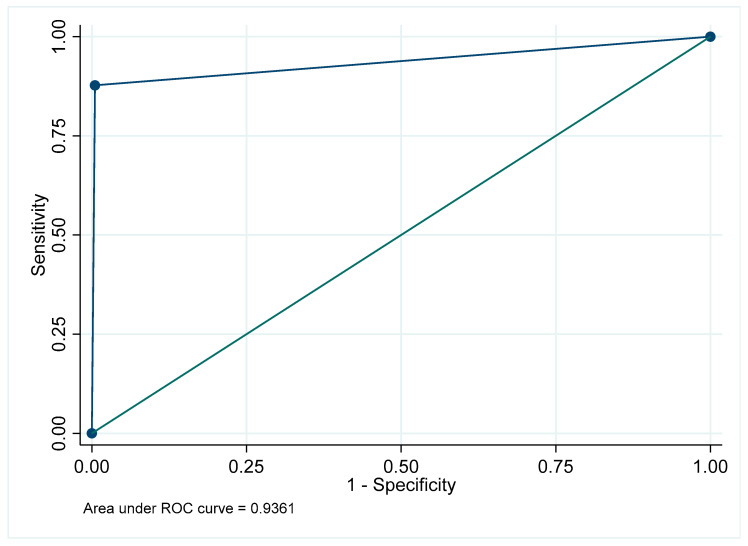
Receiver Operating Characteristic curve for retained root: Overall.

**Figure A44:**
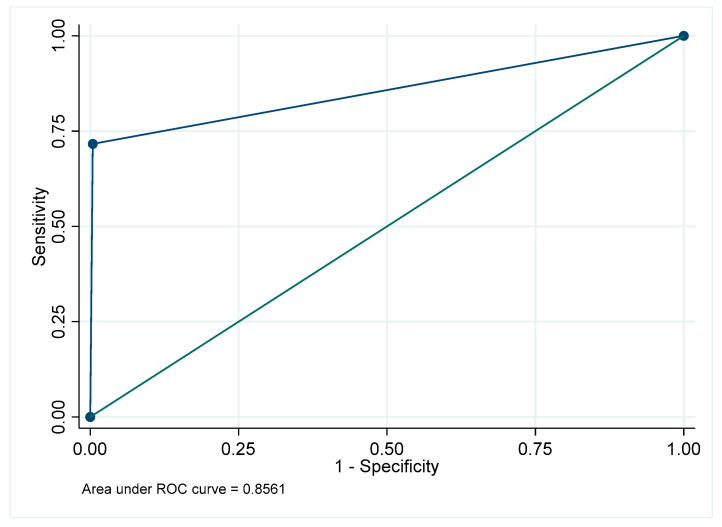
Receiver Operating Characteristic curve for caries in the maxillary posterior region.

**Figure A45:**
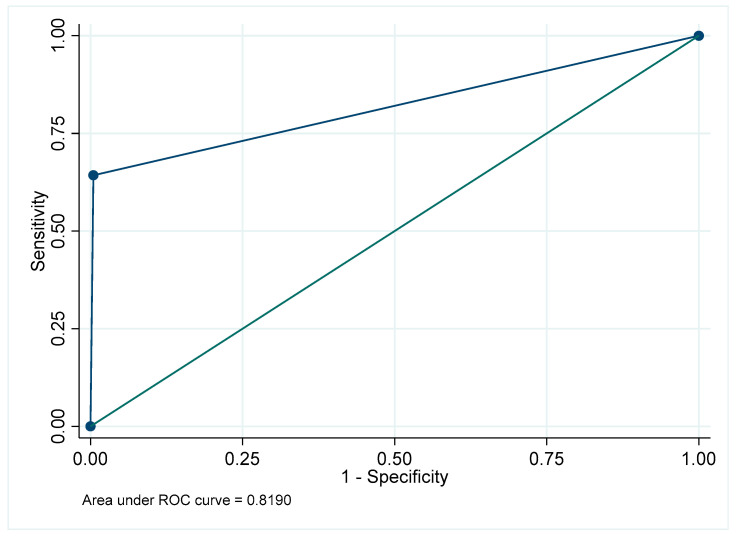
Receiver Operating Characteristic curve for caries in the mandibular posterior region.

**Figure A46:**
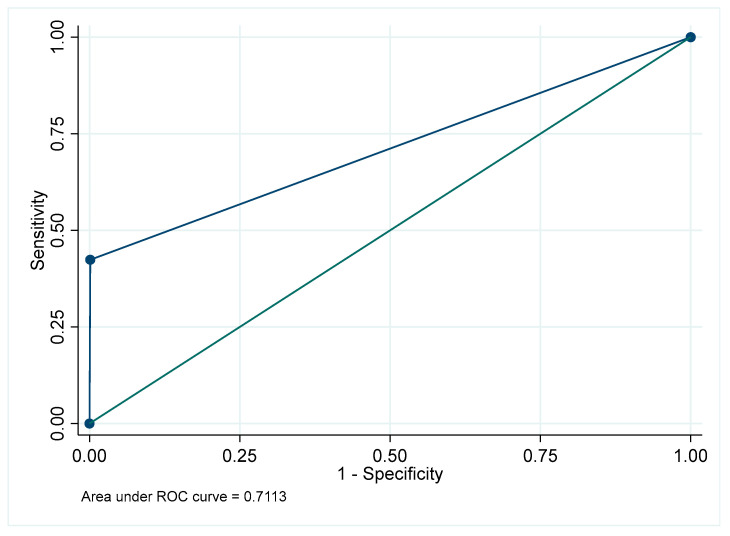
Receiver Operating Characteristic curve for caries in the maxillary anterior region.

**Figure A47:**
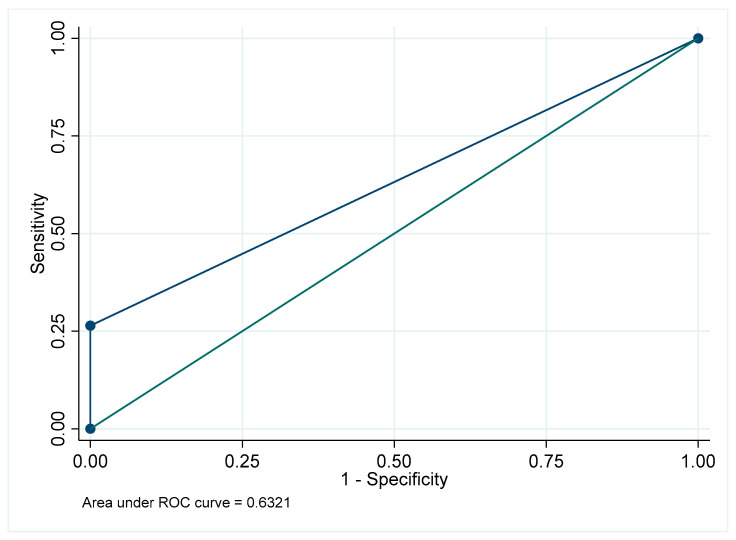
Receiver Operating Characteristic curve for caries in the mandibular anterior region.

**Figure A48:**
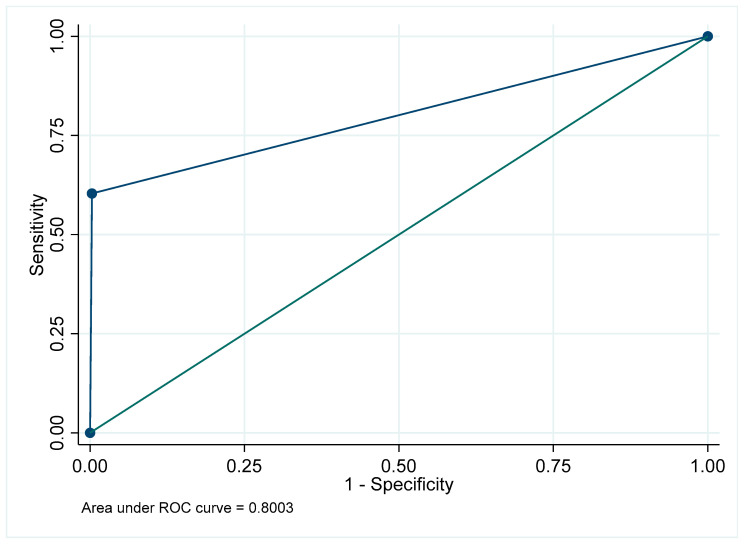
Receiver Operating Characteristic curve for caries: Overall.

## Data Availability

The original contributions presented in this study are included in the article. Further inquiries can be directed to the corresponding author.
